# Pauperism in London

**Published:** 1890-01-11

**Authors:** 


### Pauperism in London.

The poor we have always with us, and the problem of dealing with the poor we have always with us likewise, and a very troublesome one it is. To prevent strictness from developing into severity, to keep generosity from breeding demoralisation, to deal justly alike by the ratepayers, who provide money, and the poor who receive it, these are tasks so difficult and so thankless that it is no wonder our boards of guardians do not succeed so perfectly as could be desired. There is hardly a body in the country who get more abuse than they, yet anyone who was present at the recent conference of Poor Law guardians at Exeter Hall must have been convinced that they include a large number both of men and women who are honestly anxious to do their duty, though differing widely as to how that duty can best be done.

The great Poor Law problem may be briefly stated. It is this: How far is the bestowal of outdoor relief justifiable? Those who favour a generous distribution accuse those who do not of selfish hardness of heart. They draw touching pictures of Darby and Joan parted from each other in their last days to dwell in separate wards of the workhouse. The picture is not wholly accurate, but it has a certain amount of truth in it. Such cases do sometimes occur; and there is no doubt that the most respectable class of the poor feel degraded by the thought of entering the workhouse. But they show a certain lack of logic, for they feel no shame at entering an almshouse provided by private charity, nor are they humiliated at receiving the ratepayers' money in outdoor relief. There is no doubt that the poor like outdoor relief. Whether it is a good thing for them is another matter; whether, indeed, it is ever advisable to make pauperism pleasant may very well be doubted. That the wave of pity which has swept over London during the last few years has had this effect is hardly to be denied. Mr. Acworth, a member of the Wandsworth and Clapham Board of Guardians, who read an interesting paper on "Poor Law Progress and Reform in the Metropolis," thus describes the result of this indiscriminate charity: "Thanks to the generous distribution of bread and hot coffee in Trafalgar-square; to the substitution of tickets for a common lodging-house in place of admission to a casual ward; to the relaxation of the discipline in those establishments themselves; and last, but perhaps not least, to the fact that the Salvation Army has set up in the business of providing a night's lodging for all comers in competition with the older and less attractive refuges, the returns of vagrancy have advanced by leaps and bounds. Then, again, the soup kitchens, which in former years were *ascriptæ glebæ*, have lately become peripatetic, and establishing themselves day by day in front of the dock gates, have set to work to enable the employés to sell their services somewhat cheaper by the help of a charitable rate in aid of wages. Finally, children's dinners in ever-increasing multitudes, penny dinners, halfpenny dinners, free dinners—all, or almost all, alike in this, that they are not maintained on a commercial basis—have given an effectual quietus to the antiquated notion that a child is a member of its parents' family, and should look to its parents for its daily food."

This severe statement is hardly beyond the mark, and it points to a serious and growing social danger. We do not mean to say that no man can receive help from another without being demoralised; but we do say that no man can get into the habit of looking to another for a certain portion of unearned daily bread without calculating on that as a part of his income, and regulating his expenses accordingly. With the poor this regulation of expenses usually means, as Mr. Acworth says, selling their labour at a cheaper rate. A workingman calculates, quite honestly, that he can do with smaller wages if his children get a free dinner at school, and are even shod gratuitously, as has been done in some districts, and if he is assured that in old age he will be provided for in a way that he does not consider humiliating. Who, then, gets the ultimate benefit of this charity? Not the workman, but the employer, to whom these small wages mean larger profits. The most aggressive instance of relief funds not benefiting the pauper was one quoted by Mr. Acworth, where the owner of poor property collected from his tenants on Monday as rent the money he had helped, as guardian, to vote them on Saturday. Such an instance as this may be rare, almost phenomenal; but the principle of it goes through a great deal of our Poor Law administration. No such intention exists in the mind of either guardian or pauper, each is acting honestly in so far as he knows; but the result is, as Mr. Bury, another speaker at the conference put it, that when Lazarus has grown old in his service, Dives supports him at the expense of the ratepayers.

There are, indeed, cases where outdoor relief is justifiable; but, harsh as it seems to say it, these are more common where the applicants are young than where they are old. Canon Blackley has for nearly twelve years been preaching strenuously, eloquently, but almost vainly, a system of national insurance, by which a small investment in youth—£10 to £12, payable from the age of eighteen to twenty-one, or even at a slower rate—would secure a small provision for old age. Is it likely that such a scheme will ever be adopted, in spite of its obvious merit of moral training in thrift as well as its material benefit, if the working classes know that they will be provided for by someone else in a way they do not consider objectionable? Unless pauperism be an unwelcome doom, it is asking too much of human nature to expect people to evade it. On the other hand, if we take the case of a young widow, burdened with a young family, outdoor relief granted for a year or two may enable her to bring up her children till they are of age to provide for themselves without breaking up the family. Cases must be judged on their individual merits, and deserve stricter enquiry and more careful consideration than they often now receive. No one would wish to inaugurate a reign of needless severity; but the indulgence which makes pauperism a regular concomitant of old age is a doubtful boon to the poor.

There can be no doubt—and the fact is the most powerful excuse for the generous dole of outdoor relief—that the horror with which the workhouse is regarded by the respectable poor has been justified in the past by maladministration, carelessness, and even cruelty. Our workhouses are by no means perfect now, and on their defects we shall touch at another time. It would be well if guardians paid more attention to the details of workhouse management, and did not invariably accept the reports of governor and matron without examination, and in many matters connected with the kitchen, nursery, etc., the interference of lady guardians is in the highest degree desirable. Still, they are on the whole healthy places where the destitute poor are decently kept and reasonably fed. But wherever a number of people are gathered together certain regulations are necessary, and there can be no doubt that these regulations are objectionable to many of the poor. They want their liberty, they say. There is no fault to find with the sentiment, but liberty must be earned, not enjoyed at someone else's expense. We do not wish to recommend harshness to the poor, but the intention of the Poor Law is the restriction of pauperism, not the encouragement of it, and it never promised more than that no one, however unworthy, should die of hunger or cold in our midst. Poor Law relief is granted from funds raised by a compulsory rate, and when any man has a certain part of his income taken from him, whether he will or no, he has a right to demand that it shall not be wasted in any way—not even to gratify the beneficent impulses of a good-natured guardian. It is not private charity; it is not meant to compete with private charity, which provides for the comfort, as well as the necessities, of the deserving poor. To lay down an absolute rule for the ever-varying circumstances of human life is wholly impossible; but in the interests of the poor themselves it is not well to make pauperism too easy for them.

Lest we should seem to be preaching a harsh doctrine, we will conclude this part of the subject by narrating an incident told by Mr. Pell, the chairman of the conference we have spoken of, which shows that the working classes themselves perceive the justice of what we have said when they themselves study the problem. Would that there were working-men guardians on every board! A gentleman in the East-end, a rich man, and a guardian, heard that there was some discontent at the severity shown by the board of which he was a member. He called together some working-men he knew, gave them a cheque for £50, and bade them investigate for themselves the cases of which there had been complaint, and, if they thought fit, expend the money he gave among them. He stipulated only that the investigation should be thorough. After some weeks they brought him back £43. "We can't spend this," they said. "We have gone through all the cases, and we have only found it possible to give away £7, and now we are sure we have done harm by that."

*(To be continued.)*

